# Insights into the membrane repair mechanism by the coiled-coil-mediated oligomerization of TRIM72

**DOI:** 10.1016/j.bbrep.2025.102308

**Published:** 2025-10-12

**Authors:** Si Hoon Park, Georg Kempf, Hyun Kyu Song

**Affiliations:** a Department of Life Sciences, Korea University, 145 Anam-ro, Seongbuk-gu, Seoul, 02841, South Korea; b Friedrich Miescher Institute for Biomedical Research, Fabrikstrasse 24, Basel, 4056, Switzerland

**Keywords:** Coiled-coil, E3 ubiquitin ligase, Hendecad repeat, Membrane curvature, MG53, Oligomerization, TRIM72

## Abstract

TRIpartite Motif-containing 72 (TRIM72, also known as MG53), a RING-type E3 ubiquitin ligase, is critical for plasma membrane repair. Like other TRIM family proteins, TRIM72 has a conserved architecture comprising RING, B-box, coiled-coil, and C-terminal PRY-SPRY domains. While the coiled-coil domain mediates homo-oligomerization, its specific contribution to the membrane repair machinery remains unclear. In this study, we characterized the structural and dynamic properties of the TRIM72 coiled-coil domain, aiming to elucidate its contribution to membrane association. Small-angle X-ray scattering and molecular dynamics simulations revealed that the coiled-coil domain exhibits significant flexibility, including directional movements perpendicular to the membrane. Cryo-electron microscopy further demonstrated that coiled-coil-mediated oligomerization facilitated the tethering of adjacent liposomes. These findings highlight the role of the coiled-coil domain in supporting higher-order assembly on membranes, providing mechanistic insights into the TRIM72-mediated membrane repair.

## Highlights

• TRIM family proteins possess a characteristic hendecad-repeat coiled-coil structure.
• The peripheral region of the coiled-coil domain of TRIM exhibits extreme flexibility.
• The coiled-coil domain is involved in its oligomerization on the membrane.
• The coiled-coil-mediated oligomerization facilitates the tethering of adjacent membranes.

## Graphical abstract

**Figure:**
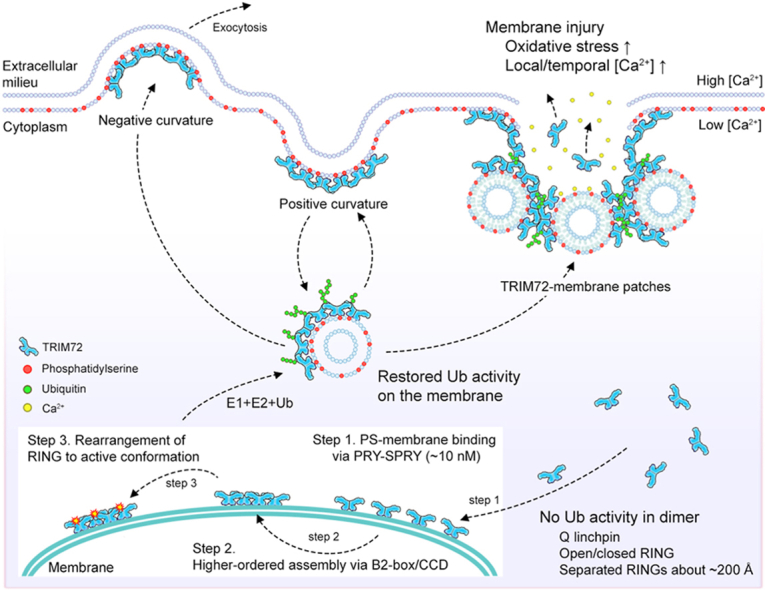

## 1 Introduction

Plasma membrane injury is a critical cellular defect that requires rapid repair to maintain the integrity of the cytoplasmic compartment and to protect it from the extracellular environment. In muscle tissue, such damage occurs frequently owing to both mechanical and metabolic stresses, not only during physical activity but also at rest [1]. Damaged membranes are promptly resealed by an evolutionarily conserved membrane repair machinery [2]. However, impairment of this process leads to a variety of human diseases, including muscular dystrophy and cardiomyopathy [2]. Since the initial discovery of membrane resealing, several models have been proposed to explain its underlying mechanisms: (1) the patch model, (2) tension reduction model, (3) exocytosis/endocytosis-mediated repair model, and (4) ESCRT-mediated budding model. Although recent research has provided substantial biochemical evidence supporting each of these mechanisms [3], the precise molecular basis underlying membrane resealing remains incompletely understood.

**Fig. 1:**
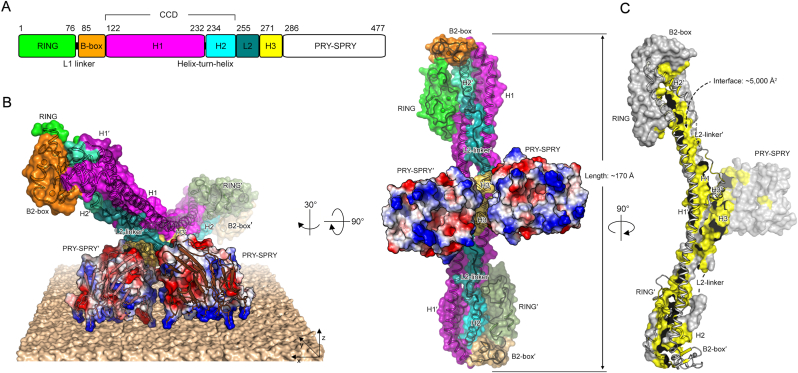
| Structure of TRIM72. **A.** Domain organization of TRIM72. Each domain is depicted as follows: RING (green), B-box (orange), H1 (magenta), H2 (cyan), L2 (teal), H3 (yellow), and PRY-SPRY (white). Residues and small motifs are annotated on the top and bottom, respectively. **B.** Overall structure of TRIM72 represented by a surface and ribbon diagram. The color of each domain corresponds to the domain architecture in panel (**A**). The second protomer is labeled with a prime symbol (′) and depicted in less vibrant colors. PRY-SPRY domains are presented with an electrostatic potential surface (positive and negative charges are indicated in blue and red, respectively). Positively charged residues are represented as a stick model. The proposed membrane-binding mode of TRIM72 is also presented. **C.** Buried surface area of a protomer, illustrated in yellow color, measuring approximately 5000 Å^2^. For clarity, the other promoter is presented as a ribbon diagram.

E3 ubiquitin (Ub) ligases play critical roles in diverse cellular processes, including signal transduction, DNA damage repair, and membrane trafficking [4]. They are classified into three different types depending on the mechanism of Ub transfer: the Really Interesting New Gene (RING), Homologous to E6-AP Carboxyl Terminus (HECT), and RING-Between-RING (RBR) [5]. Notably, the RING class is the predominant type. One of the most abundant families of RING-type Ub ligases is the TRIpartite Motif (TRIM)-containing proteins, which include more than 80 members among the approximately 600 Ub ligases identified in humans [6]. TRIM72, also known as mitsugumin 53 (MG53), is mainly expressed in muscle tissues [7]. It serves as a key initiator of plasma membrane repair following acute membrane damage, such as ischemia or reperfusion injury [8,9]. TRIM72 recruits intracellular vesicles to damaged sites, where they accumulate and form patches that help reseal the disrupted plasma membrane [9]. Its C-terminal PRY-SPRY domain binds directly to negatively charged phospholipids, particularly phosphatidylserine [10,11]. Upon binding, TRIM72 assembles into higher-order oligomers through cooperative interactions involving the B-box/B-box and coiled-coil/coiled-coil domains (Fig. 1A). The coiled-coil domain in TRIM proteins plays a role in protein-protein interactions, as shown in TRIM29. The flexible coiled-coil nature allows TRIM29 to interact promiscuously with NEMO, STING, and the membrane protein PERK [[12], [13], [14]]. However, recent data suggest that the coiled-coil domain of TRIM72 may directly bind to the membrane, contributing to membrane repair [11]. However, owing to its inherent flexibility and non-canonical architecture, the precise structural role of the coiled-coil domain remains poorly understood. Therefore, elucidating its structural details is essential for understanding the molecular mechanisms by which TRIM72 facilitates membrane repair.

In the present study, we aimed to clarify the role of the TRIM72 coiled-coil domain in higher-order assembly and membrane repair by investigating its structural properties. Using small-angle X-ray scattering (SAXS) and molecular dynamics (MD) simulations, we found that the coiled-coil domain exhibits distinct directional flexibility, including perpendicular motion relative to the membrane surface. Our analysis of multiple crystal structures with sequence alignments demonstrated that this perpendicular movement is conserved across TRIM family proteins containing RING, B-box, and coiled-coil (RBCC) domains (Fig. 1A). Further cryo-electron microscopy (cryo-EM) data revealed that coiled-coil-mediated oligomerization of TRIM72 promotes the tethering of adjacent liposomes. Overall, this study provides mechanistic insight into how the coiled-coil domain of TRIM72 mediates vesicle patch formation during membrane repair.

## 2 Materials and methods

### 2.1 Small-angle X-ray scattering

Samples were prepared as previously described [10]. *Ab initio* model determination was performed using DAMMIN software in ATSAS online (https://www.embl-hamburg.de/biosaxs/atsas-online/). The DAMMIN models were evaluated using DAMSEL, superimposed with DAMSUP, and averaged using DAMAVER. The averaged SAXS envelope models were superimposed onto the crystallographic model of mouse TRIM72 ΔRING (PDB entry 7XV2) using SUPCOMB in the ATSAS software package [15]. Detailed parameters and results are described in Supplementary Table S1.

### 2.2 Cryo-EM

For the cryo-EM experiments, TRIM72-80 mol% PS-SUV complexes were loaded onto glow-discharged Quantifoil grids and frozen in a plunge freezer with a Vitrobot (Thermo Fisher Scientific, Waltham, MA, USA). Cryo-EM images were collected at the Korea Basic Science Institute (Daejeon, South Korea) using a Titan Krios transmission electron microscope (Thermo Fisher Scientific) operated at 300 kV and recorded with a Falcon 3 EC direct electron detector (Thermo Fisher Scientific). Detailed sample preparation and cryo-EM data collection methods used have been previously described [10].

### 2.3 MD simulations

The AlphaFold 3 prediction of the human TRIM72 dimer was used as the initial starting model [16,17]. A simulation system was established with the openMM framework (version 8.1.1) [18] using the Amber14 forcefield [19] and TIP3P-FB explicit solvent model [20]. Periodic boundary conditions were applied, and particle mesh Ewald summation was used with a cutoff of 1 nm. Hydrogen bonds were constrained, and the Langevin middle integrator was used at a temperature of 300 K, a friction coefficient of 1/ps, and a step size of 0.002 ps. The protein model was energy-minimized and equilibrated over 10,000 steps (0.02 ns). For production, the system was coupled to a Monte–Carlo Barostat with pressure and temperature targets of 1 bar and 300 K, respectively. The simulation was run for 15,000,000 steps (30 ns). The MD analysis toolkit was used to calculate root-mean-square deviation (RMSD) and root-mean-square fluctuation (RMSF) values over the trajectory [21].

### 2.4 Multiple sequence and structure analyses

Sequences from human TRIM family proteins were aligned using Clustal Ω [22] and further analyzed using BioEdit software [23]. The logo graph was generated via WebLogo (https://weblogo.berkeley.edu/logo.cgi). Structural analysis was performed using the PISA server in the CCP4 suite [24]. Structure-based sequence alignment was conducted using PROMALS3D [25], with minor modifications. All structure figures were prepared with *PyMOL* (Schrödinger, LLC). The membrane model in Fig. 1B was generated based on the POPS/POPC bilayer [26].

## 3 Results

### 3.1 Overall structure of TRIM72 with an unusual coiled-coil domain

TRIM72 exhibits the typical domain architecture of TRIM proteins, featuring an RBCC domain followed by a distinctive PRY-SPRY domain (Fig. 1A). Dimeric TRIM72 adopts a bird-like shape with broad, wing-like extensions (Fig. 1B and C). The core structure consists of H1:H1′ helices, heptad H3:H3′ helices (where the “prime” symbol indicates the second protomer of the dimer), and a pair of PRY-SPRY domains that are structurally rigid in our dimer models, which is a feature unique to the TRIM superfamily [[27], [28], [29], [30], [31]].

**Fig. 2:**
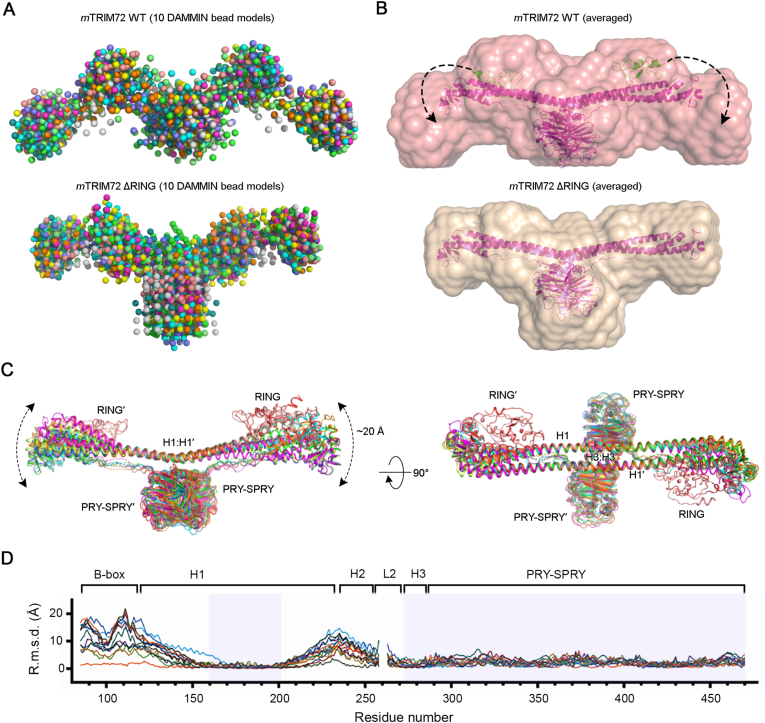
| Flexibility of the RBCC domain of TRIM72. **A.***Ab initio* DAMMIN bead models of full-length TRIM72 WT (upper) and TRIM72 ΔRING (lower). Ten bead models of each protein are superimposed. **B.** Small-angle X-ray scattering molecular envelope models of TRIM72 (upper) and TRIM72 ΔRING (lower). High-resolution crystal structures of the TRIM72 ΔRING dimer (magenta) and RING (green) are superimposed and presented as ribbon diagrams. Each envelope model was generated from an average of 10 bead models in panel (**A**). The additional volume at both ends of the full-length TRIM72 is expected to represent the RING domain in its position when in an open conformation in solution. **C.** Conformational movement of the coiled-coil. Superposition of all eight determined structures reveals that the core formed by the central H1:H1′ and H3:H3′ helices and a pair of PRY-SPRY domains is highly rigid. In contrast, the peripheral H1 and H2 helices and B-box domain are highly flexible with a maximum movement of 20 Å. Notably, the coiled-coil moves in only one direction, moving closer to and farther from the membrane. **D.** Plot of the RMSD of equivalent Cα atoms between full-length and various models as a function of residue number. The rigid regions used for structural superposition are shown in transparent cyan in the Cα-RMSD plot.

To determine the conformation of the RING domain in solution, we performed SAXS analysis (Fig. 2A). The crystal structures were fitted to SAXS molecular envelopes, revealing the flexible nature of the peripheral region of TRIM72, particularly the RING domain (Fig. 2B). The RING domain appeared to be highly mobile and was not visible in the electron density map of most full-length crystal forms [10]. In contrast to the RING domain, the core region, comprising two PRY-SPRY and adjacent H1:H1′ coiled-coil domains, was highly rigid (Fig. 2C). Notably, we observed large structural changes at both ends of the coiled-coil, characterized by movement strictly in the vertical (“up-and-down”) direction, without any lateral (“side to side”) movements. From the well-matched hendecad repeats to the flexible ends of the coiled-coil, the conformational change gradually increased, resulting in an approximate 20 Å displacement at the ends (Fig. 2D).

### 3.2 Flexible nature of the RBCC domain

**Fig. 3:**
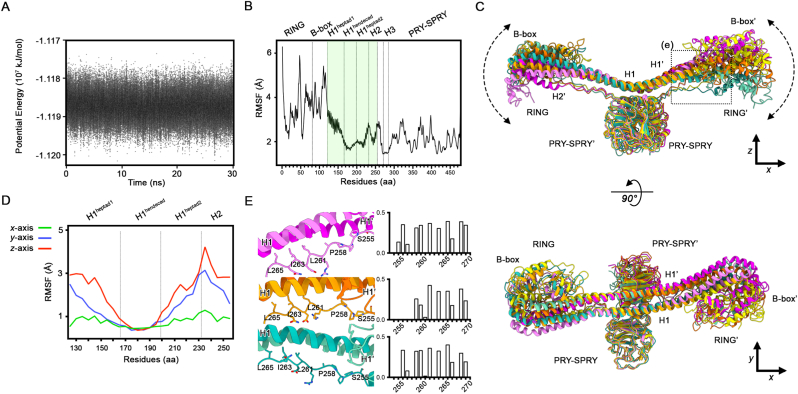
| Molecular dynamics simulation of TRIM72. **A.** Solute potential energy over a 30-ns trajectory. **B.** Domain-specific difference of the root-mean-square fluctuations (RMSF) of TRIM72 during the 30-ns trajectory. The residues of the coiled-coil domain used for calculating RMSF values along the Cartesian axis are highlighted in transparent green. **C.** “Up-and-down” movement of TRIM72 at the different time points during the MD simulation. Models are extracted at 0 ns (yellow), 10 ns (magenta), 20 ns (orange), and 30 ns (aquamarine) for clarity. The ribbon diagrams are presented from the side (top panel) and top views (bottom panel). **D.** RMSF calculation of the coiled-coil domain along the Cartesian axis. Each axis is illustrated in panel (**C**). **E.** Dynamic interactions between the dimeric heptad and the L2 linker. Ribbon diagrams (left) and buried surface area plots (right) for each model in the MD simulation trajectory are depicted in panel (**C**).

To investigate the flexibility of the coiled-coil domain, we conducted MD simulations on the TRIM72 dimer (Fig. 3). During these simulations, the system was equilibrated over 500-ps, reaching an energy minimum of approximately −118.7 MJ mol^−1^ (Fig. 3A). The RMSF profile revealed that the RING and B-box domains exhibited high mobility, as they are structurally separate from the coiled-coil domain (Fig. 3B). The RMSF values gradually decreased across the first heptad region of H1 and stabilized within the hendecad repeat of the same helix, forming part of a rigid structural core together with H3 and the PRY-SPRY domains, as observed in crystal structures [10,32]. Notably, this structural core displayed the lowest RMSF values and remained superimposable throughout the MD simulation (Fig. 3C and D).

In contrast to the rigid core, the proximal region of the coiled-coil domain exhibited a directional movement during the simulation. Motion analysis along arbitrarily defined Cartesian axes revealed the greatest fluctuations along the z-axis, moderate movement along the y-axis, and minimal displacement along the x-axis, which runs parallel to the coiled-coil domain (Fig. 3C and D). Notably, the first heptad of H1 exhibited the most pronounced movement along the z-axis. To further investigate this directional movement, we examined the interaction between the first heptad of H1 and the L2 linker. The L2 linker, which was frequently unresolved in the crystal structure due to its flexibility, dynamically interacted with the dimeric interface between the first heptad of H1 and the second heptad of H1′ from the opposite promoter (Fig. 3E). These transient contacts, mediated by both hydrophobic and hydrophilic residues, alternated between attachment and detachment depending on the L2 linker conformation. This dynamic behavior might contribute to the overall flexibility of the coiled-coil domain. Taken together, these results indicated that the RBCC domain exhibits significant flexibility, driven by its modular organization and dynamic interdomain interactions.

### 3.3 Comparison of coiled-coil domains among TRIM family proteins

To assess whether the vertical (“up-and-down”) motion of the coiled-coil is conserved across the TRIM superfamily, we compared available TRIM coiled-coil structures (Supplementary Fig. S1A) [[27], [28], [29], [30]]. These structures commonly display both heptad (*a*-*g*) and hendecad (*a*-*k*) repeat patterns, although some deviations were noted in the heptad repeats of the H2 and H3 helices. In particular, the buried hydrophobic positions in H1 helices were well aligned in TRIM coiled-coil structures (Supplementary Fig. S1B). Notably, structural superimposition revealed a similar “up-and-down” of displacement coiled-coil domains, consistent with our structural alignment and MD simulation of TRIM72 (Supplementary Fig. S1C). To expand this comparison, we aligned 64 human TRIM coiled-coil domains, revealing conserved hydrophobic residues at repeat positions (Supplementary Fig. S2 and S3). Dimeric coiled-coils were formed via H1–H1′ interactions, while trimeric interfaces involved H1, H1′, and H2′ helices (Supplementary Fig. S4A). The *a* and *d* positions of the first dimeric heptads of H1 were relatively hydrophilic, despite being buried in crystal structures (Supplementary Fig. S1). This feature may contribute to the local flexibility at the dimeric interface. Supporting this notion, RMSD analysis of TRIM72 structures revealed increased variability near residue 150, toward the N-terminal peripheral region of the coiled-coil (Fig. 2D). These findings suggest that the buried hydrophilic residues at the dimeric interfaces may facilitate the directional motion of the coiled-coil domain, likely representing a conserved feature among TRIM proteins.

### 3.4 Helix packing of the four helical bundle

The dimeric hendecad repeats in the H1 helix were the most rigid parts shown in the RMSD analysis among all determined TRIM72 structures (Fig. 2C and D), as well as in comparison with the structurally characterized TRIM coiled-coil domains (Supplementary Fig. S1C). The core hendecad region of TRIM72 forms a unique tetrameric hendecad/heptad helix packing structure compared to that of other TRIM proteins (Supplementary Fig. S4). The H3 helix forms an antiparallel dimer with the H3’ helix of the other protomer and further packs against four-helical bundles at the middle of the antiparallel H1:H1’ dimer (Supplementary Fig. S4B). A tetrahelical bundle consisting of the H3:H3’ heptad and H1:H1’ hendecad dimers at an angle of 50°, which is commonly observed for α-helix packing [33], was present (Supplementary Fig. S5), although the corresponding bundles in other TRIM coiled-coils showed different angles (Supplementary Fig. S4C–G). The stable four-helical bundle structure of TRIM72 is critical for its function, as confirmed by the deletion of the H3 helix, which lacks a membrane-binding affinity [10]. The angles between hendecad H1:H1’ and heptad H3:H3’ are variable: from 0 to 90° among the TRIM-family proteins (Supplementary Fig. S4B–G). As shown in the domain architecture of TRIM-family proteins, additional functional domains exist after the H3 helix. Therefore, the orientation of the H3:H3’ heptad might be important for the physiological role of TRIM-family proteins.

### 3.5 Higher-order TRIM72 assembly mediates membrane tethering via the coiled-coil domain

**Fig. 4:**
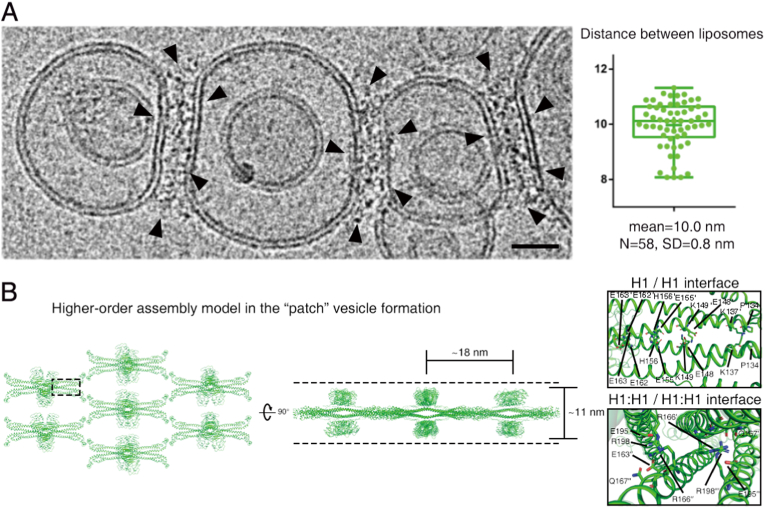
| Higher-order TRIM72 assembly on the lipid membrane. **A.** Cryo-electron micrograph showing proteoliposomes with the TRIM72 oligomer on phosphatidylserine-SUVs. TRIM72-TRIM72 contacts were observed in the peripheral region between separate liposomes (left). Black scale bars indicate 20 nm. Box plot of the distance between the TRIM72 oligomer-bound liposomes (right). Mean and median values (small and long bars in the box, respectively) display the first and third quartiles (upper and lower ends of the box, respectively). Each value (dot) is represented with whiskers from minimum to maximum. **B.** The molecular model of vesicle-patching assembly. Top view (left) and side view (middle) rotated by 90°. The model is derived from the crystal packing of TRIM72 WT (7XYY). Notably, the distance between the patched liposomes is approximately 10 nm on the cryo-EM image, which is close to the height of the oligomer at approximately 11 nm in the middle of panel (**B**). The dashed lines indicate two sides of the membrane surface.

We have previously demonstrated that TRIM72 assembles into a higher-order oligomeric layer on negatively charged membranes [10]. This oligomerization is mediated by homotypic interactions between B-box/B-box domains and coiled-coil/coiled-coil domains, promoting ubiquitylation and membrane binding. Unexpectedly, during the examination of TRIM72-bound liposomes in cryo-EM in the present study, we observed that TRIM72 oligomers frequently bridged adjacent liposomes, forming distinct tethering structures (Fig. 4A). The average distance between tethered liposomes was approximately 10 nm (Fig. 4A), exceeding the approximately 7 nm height of a single TRIM72 oligomer layer. This observation suggests a previously uncharacterized oligomeric pattern.

To gain further insight into this configuration of the TRIM72 oligomer, we examined the crystal packing of TRIM72. Among six distinct packing interfaces (PDB entries 7XYY, 7XZ0, 7XZ1, 7XZ2, 7XYZ, and 7XV2), crystal packing of TRIM72 WT (PDB entry 7XYY) revealed direct contacts between coiled-coil domains (Fig. 4B). In this arrangement, two PRY-SPRY dimers were oriented in opposite directions, potentially enabling simultaneous engagement with membranes on both sides. The distance between the opposing PRY-SPRY domains was approximately 11 nm. Given the perpendicular flexibility of the coiled-coil domain, this bilayer-like higher-order assembly may become compacted, closely matching the approximately 10 nm spacing observed in cryo-EM. Overall, these findings suggest that TRIM72 forms a coiled-coil-mediated bilayer assembly capable of tethering adjacent membranes, supporting its role in membrane repair [7,34,35].

## 4 Discussion

In the present study, our structural alignments and MD simulations revealed that the TRIM72 coiled-coil domain exhibits directional flexibility, primarily through an up-and-down (vertical) motion. This behavior is driven by the structural contrast between the rigid hendecad repeats in the H1 helix, characterized by their unique periodicity (∼3.67 residues per turn), and the more flexible heptad repeats in the H1 and H2 regions. This flexibility is further enhanced by the dynamic interactions with the L2 linker, while the linear hendecad helix involves motion along a vertical axis. Notably, this behavior is consistent with recent cryo-EM analyses of TRIM72 [36], highlighting that vertical flexibility is a conserved and functionally relevant feature of TRIM72 RBCC domains responsible for ubiquitin transfer and oligomerization.

The ubiquitylation activity and oligomerization of TRIM72 are tightly coupled with its membrane-binding capacity. We previously proposed an activation model in which RING domains dimerize during higher-order assembly on the phospholipid membranes [10]. Notably, TRIM72 oligomers have been observed on both concave and convex membrane surfaces [10], suggesting that the flexibility of the coiled-coil domain enables adaptation to a wide range of membrane curvatures. However, it remains unclear whether TRIM72 actively remodels membranes, as observed in Bin/Amphiphysin/Rvs (BAR) domain-containing proteins that sense and reshape curved membranes [37]. Notably, we observed that TRIM72 assemblies often localize to the inner surface of liposomal membranes, potentially owing to their internalization by larger liposomes. However, the functional relevance of this event remains to be determined.

In addition to the single-layered higher-order assembly of TRIM72, we now propose a novel mode of assembly in which TRIM72 promotes the tethering of adjacent membranes. Unlike lateral assemblies confined to a single membrane plane, this tethering assembly appears to arise from interactions between TRIM72 oligomers anchored to separate membranes. Based on our crystallographic packing analysis, we suggest that this tethering assembly is mediated by direct coiled-coil domain interactions, bridging the TRIM72 oligomer across opposing membrane surfaces. While our findings support the vesicle patch model during membrane repair, high-resolution structural data are required to elucidate how TRIM72 coordinates the membrane repair process at the molecular level.

## CRediT authorship contribution statement

**Si Hoon Park:** Writing – review & editing, Writing – original draft, Validation, Methodology, Investigation, Data curation. **Georg Kempf:** Visualization, Methodology, Formal analysis, Data curation. **Hyun Kyu Song:** Writing – review & editing, Writing – original draft, Supervision, Project administration, Funding acquisition, Conceptualization.

## Declaration of competing interest

The authors declare no competing interests.

## Data Availability

Data will be made available on request.
