# Supplementary material for: Insights into the membrane repair mechanism by the coiled-coil-mediated oligomerization of TRIM72
Source: Biochem Biophys Rep. 2025 Oct 12;44:102308. doi: 10.1016/j.bbrep.2025.102308 (PMC12549529; doi:10.1016/j.bbrep.2025.102308)
Supplement: Multimedia component 1 [file mmc1.pdf]

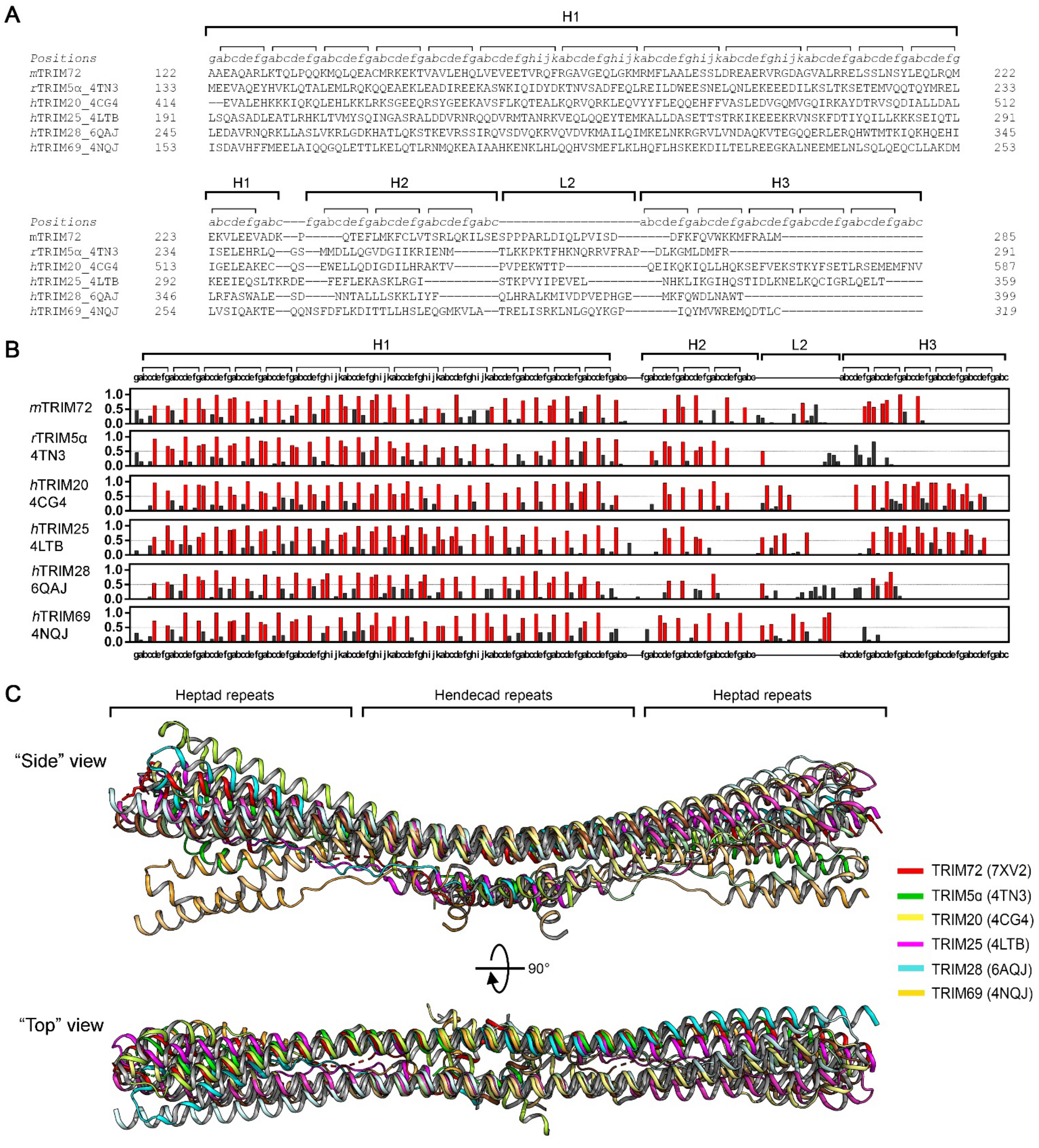

Fig. S1| Structural alignments of the TRIM coiled-coil domains.

A. Structure-based sequence alignment of TRIM coiled-coil domains. Subdomains are annotated by thick brackets at the top (H1, H2, L2, and H3). The heptad (*a*-*g*) and hendecad (*a*-*k*) repeat positions are indicated by thin brackets above the amino acids. TRIM proteins and their PDB codes are indicated in front of the sequences with their species (*m*, mouse; *h*, human; *r*, rhesus). **B.** Buried accessible surface area (ASA)/total ASA plots. The red bars indicate residues for which more than half of the ASA is buried within the interfaces. **C.** Superimposition of the TRIM coiled-coil. Four-hendecad helices of each dimer were aligned using the CE algorithm. Each coiled-coil domain is presented in the ribbon diagram and colored

as follows: *m*TRIM72 ΔRING, red; *r*TRIM5α, green; *h*TRIM20, yellow; *h*TRIM25, magenta; *h*TRIM28, cyan; *h*TRIM69, orange. The broad distribution of each flexible end of the TRIM coiled-coil is viewable only in the "side" view (upper) and not the "top" view (lower) obtained by a 90° rotation.

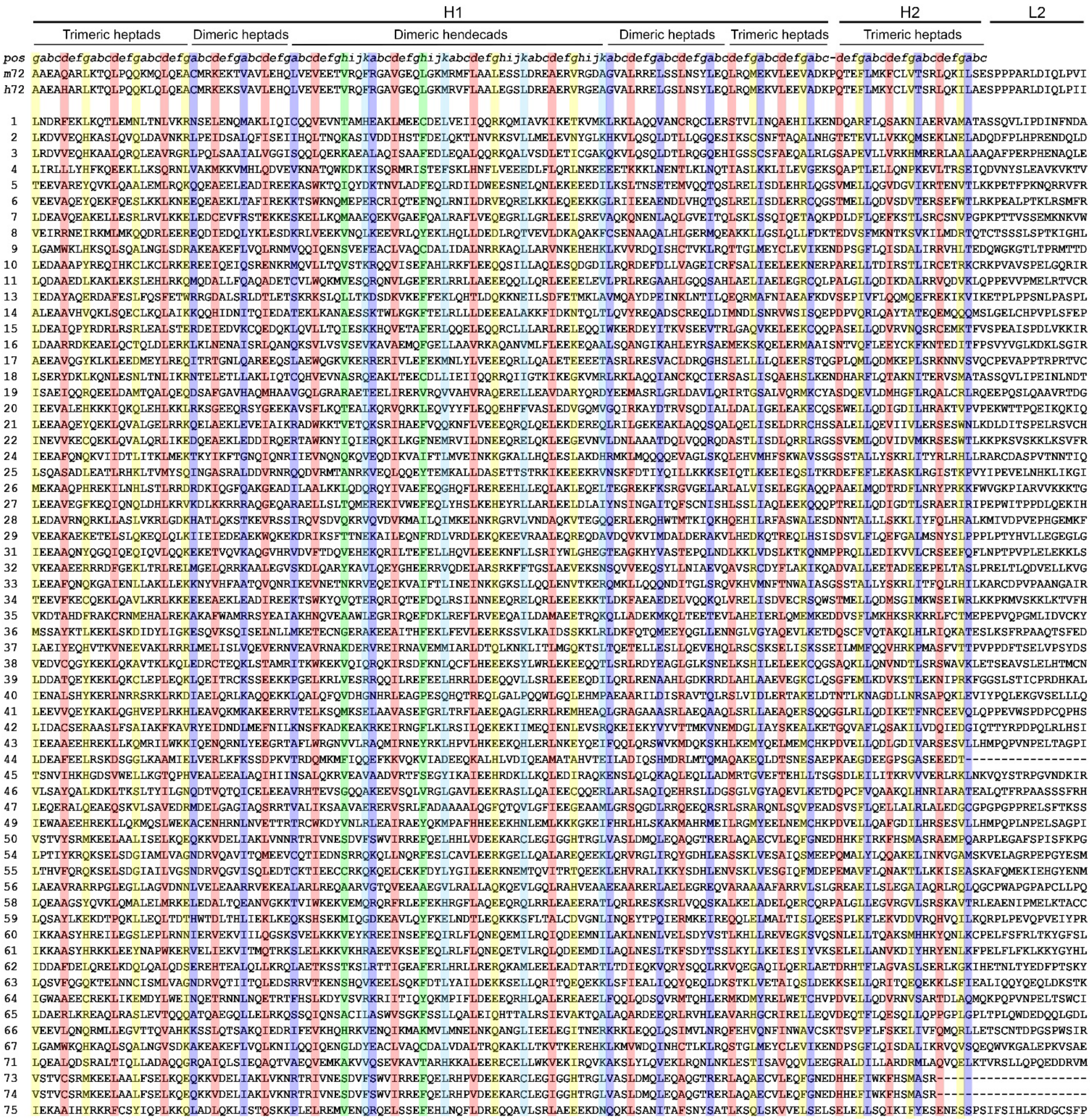

Fig. S2| Multiple sequence alignments of the TRIM coiled-coil domains.

A total of 64 *human* TRIMs were used in the analysis. Hydrophobic repeats are labeled with the following colors: *a*, blue; *d*, red; *g*, yellow; *h*, green; *k*, cyan.

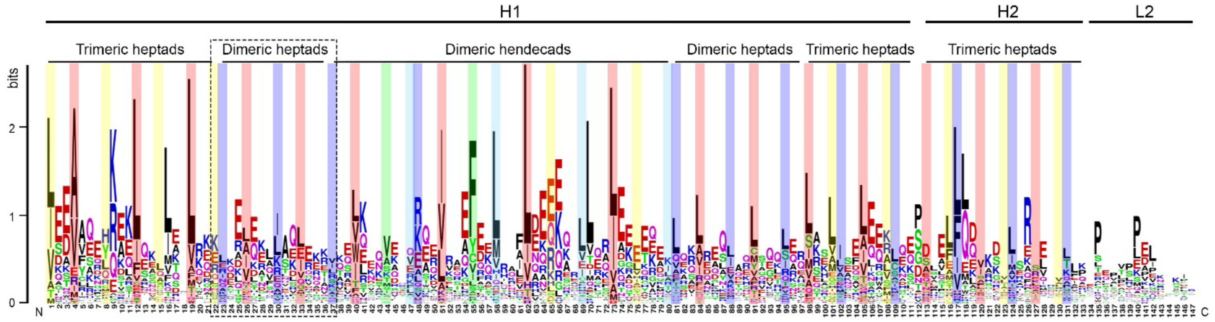

Fig. S3| Logo graph of amino acid frequency.

The dashed box, a dimeric heptad in the H1 helix, reveals no conservation in the hydrophobic repeats in the TRIM coiled-coil. Hydrophobic repeats are depicted using the following colors: *a*, blue; *d*, red; *g*, yellow; *h*, green; *k*, cyan.

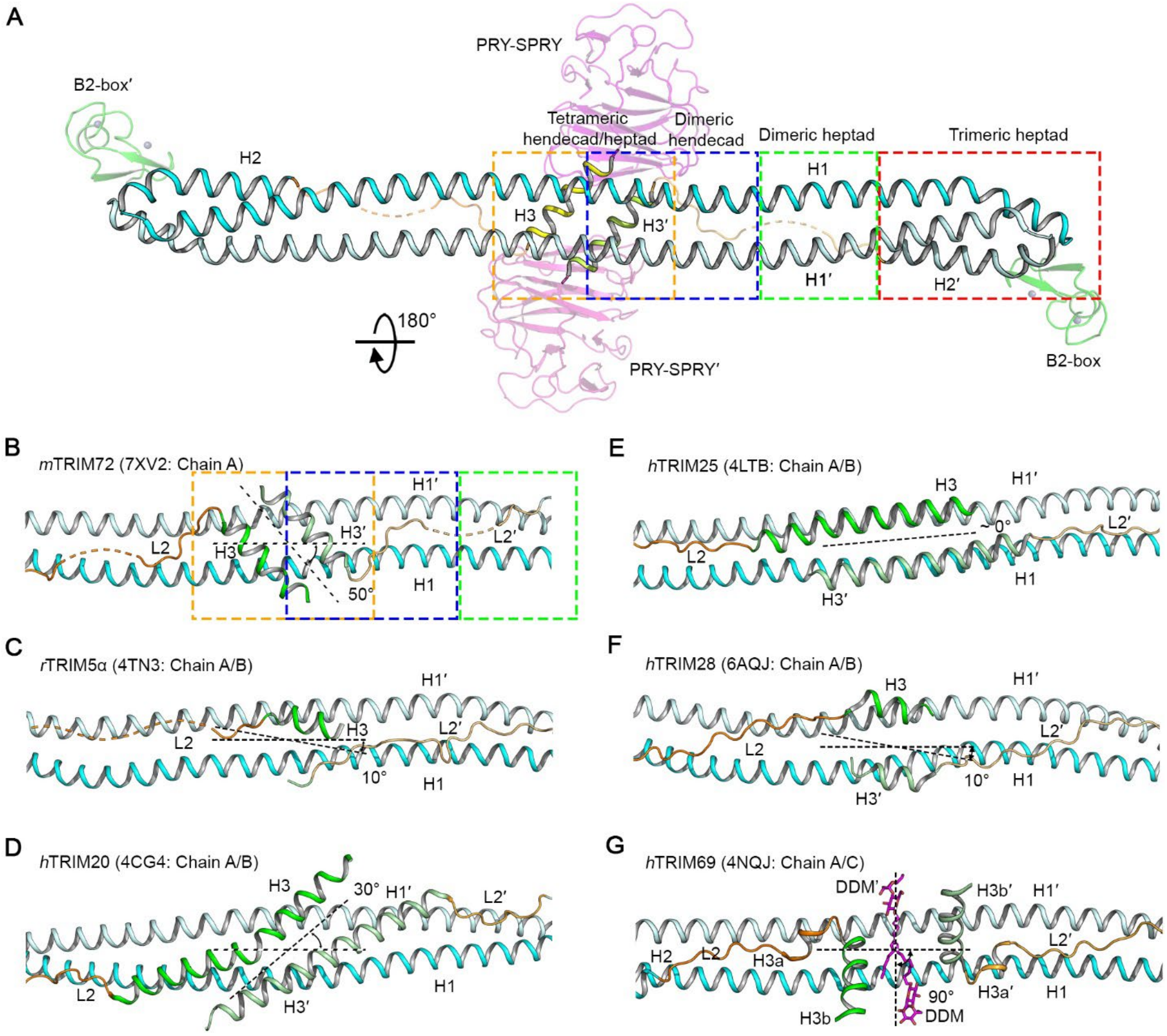

Fig. S4| Structural comparison of the coiled-coil domain in other TRIM proteins.

A. Overall structure of the *m*TRIM72 coiled-coil is presented as a ribbon diagram. The interactions are categorized as belonging to four regions: trimeric heptad (red dashed box), dimeric heptad (green dashed box), dimeric hendecad (blue dashed box), and tetrameric hendecad/heptad (orange dashed box). **B.** Structure of the coiled-coil region of TRIM72 (PDB ID: 7XV2). The view represents the 180° rotation along the horizontal axis of panel (**A**) to show the H3 and H3′ helices. **C–G.** Comparison of similar regions in other TRIM proteins (**C.** *r*TRIM5α; **D.** *h*TRIM20; **E.** *h*TRIM25; **F.** *h*TRIM27; **G.** *h*TRIM69) with the tetrahelical bundle of *m*TRIM72 (orange dashed box region of panels **A** and **B**). The H3:H3 interactions could be eliminated as a result of a deletion of the C-terminal domain in the constructs (*r*TRIM5α and *h*TRIM28) or detergent binding (*h*TRIM69). The other protomer is labeled with a prime (′) symbol. The angle between central H1:H1′ and H3:H3′ helices is indicated (**B–G**). DDM; *n*-dodecyl β-D-maltoside.

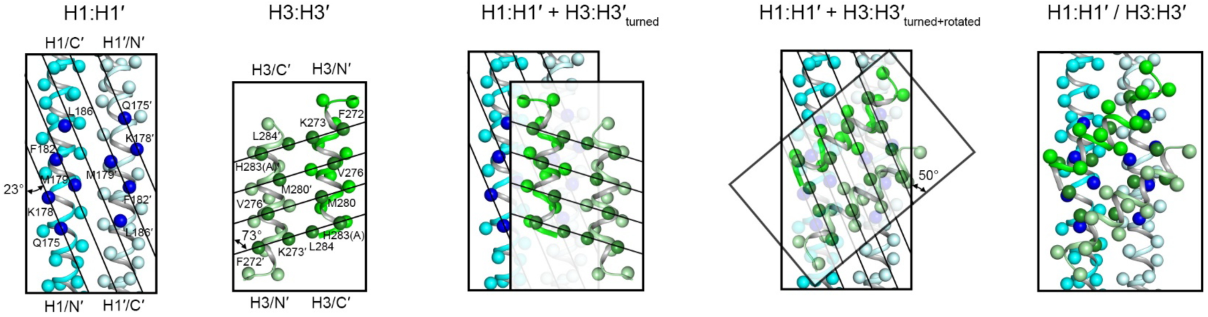

Fig. S5| Helical bundle interaction of heptad-hendecad repeats in the TRIM72 CCD. The "ridges and grooves" model of the heptad-hendecad interaction. C_β atoms are shown as spheres. The other protomer is labeled with a prime (') symbol. For H279(K) and H283(A), the residues in parentheses are from TRIM72 model (PDB entry 7XV2).

Supplementary Table 1. SAXS-derived structural parameters.

	*m*TRIM72 WT	*m*TRIM72 ΔRING
**Shape model-fitting results**		
DAMMIN (default parameters, averaged to 10 calculations)		
q range for fitting (Å^{-1})/χ^2 range	0.0079–0.1166/1.35–1.432	0.0126–0.1578/1.314–1.386
Symmetry, anisotropy assumptions	P2/none	P2/none
Constant adjustment to intensities	Skipped	Skipped
DAMSEL, *DAMSUP*, and *DAMAVER* (default parameters)		
NSD (standard deviation)	0.632 (0.028)	0.683 (0.032)
Resolution (from *SASRES*) (Å)	44 ± 3	39 ± 3
SUPCOMB (default parameters)		
NSD (normalized spatial discrepancy)	7.71	4.52
**Atomic modeling**		
Crystal structure (PDB ID)	7XYZ	7XV2
q range for all modeling	0.0056–0.2564	0.0074–0.2743
CRYSOL (with default parameters)		
No constant subtraction		
χ^2/Predicted R_g (Å)/Vol (Å)/Ra (Å)/Dro (e Å^{-3})	7.051/50.90/135,768/1.800/0.007	3.068/50.00/115,354/1.400/0.015
Constant subtraction allowed		
χ^2/Predicted R_g (Å)/Vol (Å)/Ra (Å)/Dro (e Å^{-3})	6.743/51.07/133,242/1.800/0.022	3.057/50.00/115,354/1.420/0.015
